# Deciphering the Pharmacological Mechanisms of Guizhi-Fuling Capsule on Primary Dysmenorrhea Through Network Pharmacology

**DOI:** 10.3389/fphar.2021.613104

**Published:** 2021-03-03

**Authors:** Siqin Zhang, Xinxing Lai, Xin Wang, Gang Liu, Zhenzhong Wang, Liang Cao, Xinzhuang Zhang, Wei Xiao, Shao Li

**Affiliations:** ^1^ Department of Automation, Institute for TCM-X, MOE Key Laboratory of Bioinformatics/Bioinformatics Division, BNRIST, Tsinghua University, Beijing, China; ^2^ Institute for Brain Disorders, Dongzhimen Hospital, Beijing University of Chinese Medicine, Beijing, China; ^3^ School of Pharmaceutical Sciences, Tsinghua University, Beijing, China; ^4^ State Key Laboratory of New-tech for Chinese Medicine Pharmaceutical Process/Key Laboratory for the New Technique Research of TCM Extraction and Purification/Jiangsu Kanion Pharmaceutical Co., Ltd., Jiangsu, China

**Keywords:** primary dysmenorrhea, network target, network pharmacology, pharmacological mechanisms, Guizhi-Fuling capsule

## Abstract

Guizhi-Fuling capsule (GZFLC), originated from a classical traditional Chinese herbal formula Guizhi-Fuling Wan, has been clinically used for primary dysmenorrhea in China. Nonetheless, the underlying pharmacological mechanisms of GZFLC remain unclear. The integration of computational and experimental methods of network pharmacology might be a promising way to decipher the mechanisms. In this study, the target profiles of 51 representative compounds of GZFLC were first predicted by a high-accuracy algorithm, drugCIPHER-CS, and the network target of GZFLC was identified. Then, potential functional modules of GZFLC on primary dysmenorrhea were investigated using functional enrichment analysis. Potential bioactive compounds were recognized by hierarchical clustering analysis of GZFLC compounds and first-line anti-dysmenorrhea drugs. Furthermore, the potential anti-dysmenorrhea mechanisms of GZFLC were verified through enzyme activity assays and immunofluorescence tests. Moreover, effects of GZFLC on primary dysmenorrhea were evaluated in oxytocin-induced dysmenorrhea murine model. In the network target analysis, GZFLC may act on five functional modules of pain, inflammation, endocrine, blood circulation and energy metabolism. Integrating computational and experimental approaches, we found that GZFLC significantly inhibited the writhing response and reduced the degree of uterine lesions in oxytocin-induced dysmenorrhea murine model. Furthermore, GZFLC may partially alleviate primary dysmenorrhea by inhibiting cyclooxygenase 2 (COX2) and downregulating MAPK signaling pathway. Consequently, GZFLC presented pain relief and sustained benefits for primary dysmenorrhea. This study could provide a scientific approach for deciphering pharmacological mechanisms of herbal formulae through network pharmacology.

## Introduction

Primary dysmenorrhea (PD) is defined as abnormally painful menstruation in the absence of any evident underlying pelvic pathology (Kho and Shields, 2020). Characterized by recurrent, painful, spasmodic cramping in the lower abdomen during menstruation, primary dysmenorrhea is the most common menstrual symptom among adolescent girls and young women, with the prevalence rate ranging from 41.7% to 90% (ACOG, 2018; Hu et al., 2020). As one of the most common gynecologic disorders, primary dysmenorrhea has significant effects on patients’ normal school or work, as well as quality of life (Burnett et al., 2005; Wong 2018). The pathogenesis of primary dysmenorrhea is most widely accepted and considered to be the overproduction of uterine prostaglandins (PGs) (Iacovides et al., 2015). Owing to the PGs-based etiology of primary dysmenorrhea, the current first-line pharmacological agents for primary dysmenorrhea include non-steroidal anti-inflammatory drugs (NSAIDs) and hormonal contraceptives (Kho and Shields 2020).

Despite the sufficient evidence supporting the efficacy of NSAIDs in pain relief for patients with primary dysmenorrhea, there are approximately 15% of patients not responding to, or being intolerant to NSAIDs due to many side effects (Campbell and McGrath 1999; Marjoribanks et al., 2010). Oral hormonal contraceptives are also effective for primary dysmenorrhea, however, a meta-analysis has demonstrated the long-suspected association between oral contraceptive use and the risk of venous thromboembolism (Manzoli et al., 2012). Given these disadvantages of NSAIDs and hormonal contraceptives, effective and safe alternative treatments, such as Traditional Chinese Medicine (TCM), are receiving increasing attention worldwide. TCM characterized as holistic thinking emphasizes on regulating the integrity of the human body (Li et al., 2007) and intervenes multiple aspects related to etiological factors and pathogenesis simultaneously, which has accumulated numerous valuable clinical experiences and herbal formulae through long history of development.

Guizhi-Fuling capsule (GZFLC), a Chinese patent medicine approved by China Food and Drug Administration (CFDA), is clinically used for blood stasis syndromes in gynecological diseases including primary dysmenorrhea. It originated from a classical traditional Chinese herbal formula Guizhi-Fuling Wan which was first described in a classics named the Essential Prescriptions from the Golden Cabinet (Jin Kui Yao Lue) documented in 150–219 A.D. Guizhi-Fuling capsule is the modern dosage form of Guizhi-Fuling Wan, and the dosage and ratio of each Chinese herbal medicine in Guizhi-Fuling capsule is the same as Guizhi-Fuling Wan, consisting of five herbs: *Cinnamomum cassia (L.) J. Presl* (Guizhi), *Poria cocos* (Fuling), *Paeonia lactiflora Pall* (Baishao), *Paeonia × suffruticosa Andrews* (Mudanpi) and *Prunus persica (L.) Batsch* (Taoren). Pharmacokinetic-pharmacodynamic modeling study has demonstrated that GZFLC exhibited significant therapeutic effect on primary dysmenorrhea in rat model (Cheng et al., 2018). Moreover, a randomized controlled trial showed that GZFLC significantly relieved pain and reduced the duration of pain compared with placebo during the 3-months treatment period, as well as in the 3-months follow up period, demonstrating a sustained benefit for patients with primary dysmenorrhea (Liu et al., 2013). However, the underlying comprehensive pharmacological mechanisms of GZFLC on primary dysmenorrhea remain unclear.

There are several substantial challenges in terms of deciphering pharmacological mechanisms of herbal formulae. Firstly, herbal formulae are complex chemical systems usually composed of numerous chemical compounds. Secondly, a great majority of bioactive compounds in herbs may have weak or moderate effects on multiple biological molecules, different from most western drugs designed to act on a single target selectively (Ji et al., 2009). Thus, routine pharmacologic analysis methods are difficult to systematically elucidate the mechanisms of herbal formulae. Network pharmacology, a newly developed cross-discipline, is preferable in investigation and elucidation of comprehensive mechanisms of TCM (Li et al., 2007; Hopkins 2008). The key ideas of Network pharmacology share much with the holistic philosophy of TCM (Li and Zhang 2013). TCM network pharmacology highlights a “network target, multicomponent therapeutics” approach (Li et al., 2011; Li 2015) to help elucidate the complex molecular mechanisms of traditional herbal formulae. The core theory of network pharmacology is “network target” distinct from “one target, one drug” paradigm (Li 2011; Li et al., 2011). More specifically, the core principle of network target is constructing a disease-specific biological network as the therapeutic target that herbal formulae are applied to (Li 2015). TCM network pharmacology has been successfully applied in many researches such as molecular mechanism elucidation of herbal formulae (Liang et al., 2014; Zuo et al., 2018), new bioactive compounds discovery (Qi et al., 2016), comprehensive pharmacological function of natural products identification (Zhang et al., 2018) and mechanism of action of toxic components in traditional Chinese medicines (Li et al., 2019).

**FIGURE 1:**
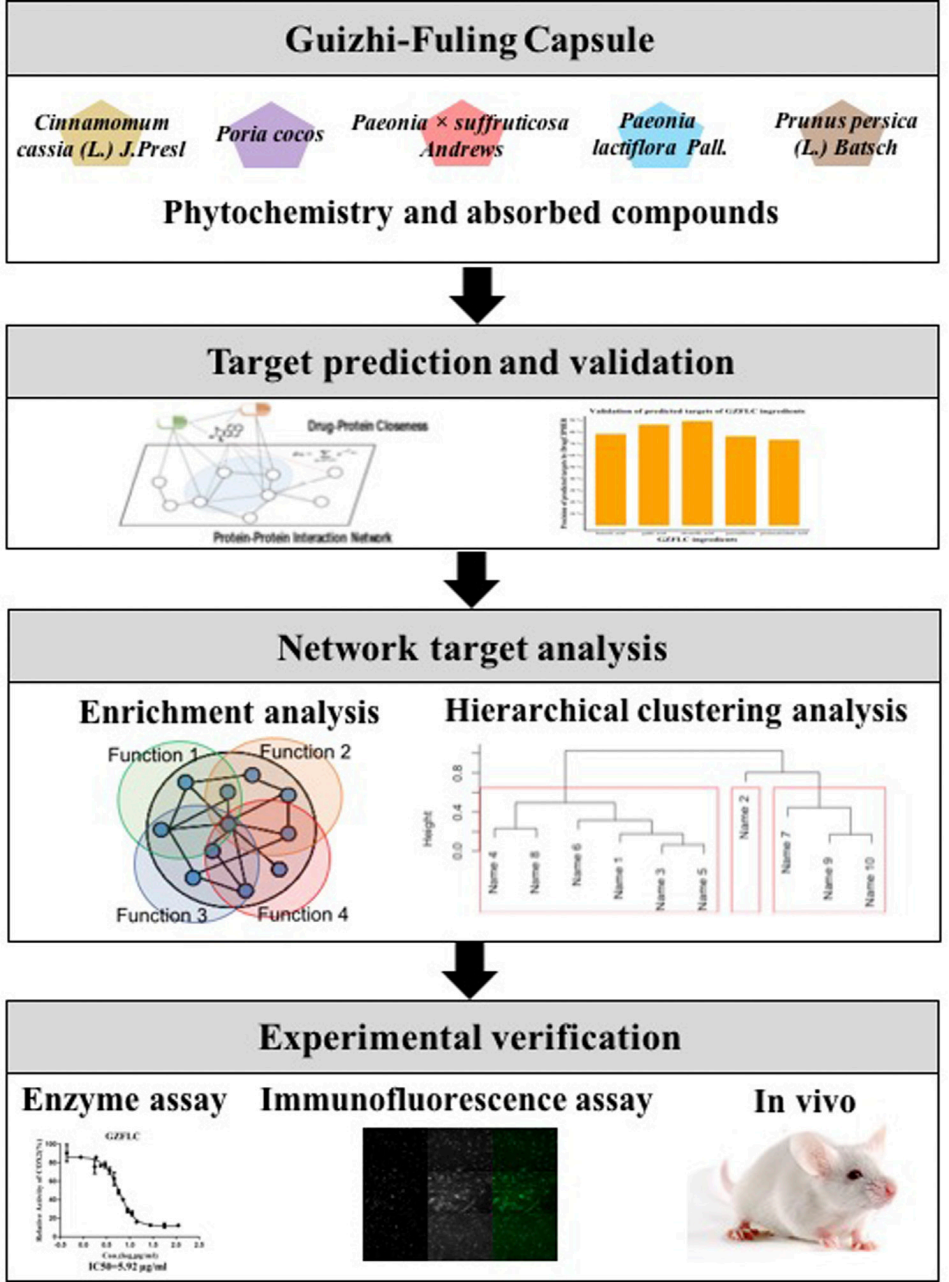
Network pharmacology analysis workflow.

In this study, we proposed an approach to investigate the underlying pharmacological mechanisms of GZFLC on primary dysmenorrhea based on network pharmacology as shown in Figure 1. First, we collected important phytochemistry and absorbed compounds of GZFLC. Second, we predicted the potential targets of GZFLC compounds utilizing a high-accuracy target prediction algorithm, and then validated the potential targets in literatures to ensure the reliability of predictions. Third, we made the network target analysis of GZFLC to investigate the pharmacological mechanisms and bioactive compounds of GZFLC on primary dysmenorrhea, including functional enrichment analysis and hierarchical clustering analysis. These network target analysis results were verified *in vitro* and *in vivo*.

## Materials and Methods

### Representative Compounds in GZFLC Collection

The dosages and ratios of GZFLC compounds could influence their intervention intensity on targets. Considering the influence of GZFLC compounds’ dosages and ratios, we selected major phytochemistry compounds and absorbed compounds of GZFLC as representative compounds to analyze GZFLC’s pharmacological mechanisms through network pharmacology. We have investigated phytochemistry compounds of GZFLC and absorbed compounds of GZFLC in plasma previously (Zhong et al., 2016a; Yin et al., 2016; Zhang et al., 2017; Yang et al., 2020). In total, 51 compounds were selected from our previous investigations of phytochemistry and absorbed compounds of GZFLC based on the following criteria: 1) the characteristic compounds were accounted for the major proportion and verified with reference substances; 2) the compounds were quantificationally recorded in each herb from Chinese Pharmacopoeia (2015) (Committee 2015); 3) each compound had specified chemical structure and Pubchem CID.

### Preparation and Analysis of GZFLC

GZFLC was provided by the company Jiangsu Kanion Pharmaceutical Co., Ltd., composed of *Cinnamomum cassia (L.) J. Presl* (20%), *Poria cocos* (20%), *Paeonia × suffruticosa Andrews* (20%), *Paeonia lactiflora Pall* (20%), *Prunus persica (L.). Batsch* (20%). The full taxonomic names of all herbs included have been fully validated using the Kew Medicinal Plant Names Service. Guizhi-Fuling capsules have established fingerprints (Wang et al., 2009; Li et al., 2012) from herb to preparation to make sure that the quality of the preparation is uniform and stable (Li et al., 2015). Guizhi-Fuling capsule is a Chinese patent medicine approved and supervised by CFDA under the same strict quality control standards. Meanwhile, Guizhi-Fuling capsules in our study have come from the same preparation. The chemical constituents analysis of GZFLC has been reported in the literature (Yang et al., 2020). The UPLC analysis of GZFLC (Jiangsu Kanion Pharmaceutical Co., Ltd. Jiangsu, China, No. 20160319) was performed on Agilent SB-RRHD C18 (100 mm × 2.1 mm, 1.8 μm) column at 30°C with a mobile phase consisting of acetonitrile and 0.02% trifluoroacetic acid for gradient elution and the flow rate of 0.2 ml/min. The detection wavelength was set at 230 nm. The representative UPLC fingerprints of GZFLC were shown in Supplementary Figure S1 in the Supplementary Materials.

### Target Predication and Validation for Compounds in GZFLC

To explore the relativity of representative GZFLC compounds and targets, the potential target profiles of compounds in GZFLC were predicted by drugCIPHER-CS (Zhao and Li, 2010), a high-accuracy algorithm developed for global prediction of compound targets. In principle, drugCIPHER-CS used a linear regression model which related chemical similarity vector between compounds in GZFLC and drugs in DrugBank (Wishart et al., 2008) to the drug-protein closeness vector based on a protein–protein interaction network to calculate the concordance score of each compound-protein pair. The concordance score was treated as the likelihood of the compound targeting the protein. Thus, the candidate proteins with high concordance scores were prioritized to be potential targets of the compound. Top 100 ranking predicted proteins of each compound were kept as potential target profiles.

To validate the target prediction of compounds, literature mining method was used to obtain the co-occurrence results of biomolecules and each compound. The biomolecules related to each compound from literature mining were manually examined to delete the false positive results and then considered as the reported targets. Comparing the reported targets with the predicted targets via direct mapping and indirect link, the reliability of target prediction was measured by $\frac{\left|\text{The predicted targets related to the reported targets }\right|}{\left|\text{The predicted targets}\right|}*100\%$.

### Holistic Target Prediction for GZFLC and Each Herb

To identify potential targets regulated by GZFLC or herbs, predicted proteins ranking top or appearing in the target profiles of many compounds in GZFLC or herbs were hypothesized to be important in the pharmacological effects of GZFLC or herbs. Based on this hypothesis, the potential targets of GZFLC or herbs were considered as significantly frequently occurring targets (*p* < 0.05) by comparing the number of occurrences of a target protein in the target profiles of all compounds in GZFLC or herbs to that in random background (Liang et al., 2014). A Poisson binomial statistical model was used to obtain the random background distribution and represented as:

$$P\left(K=k\right)=\sum_{A\in F_{k}}\prod_{j\in A}p_{i}\prod_{j\in A^{c}}\left(1-p_{j}\right).$$

P(K=k) is the probability of a certain target in  k compounds’ target profiles, $F_{k}$ is the set including all sets containing k compounds, A is an element of $F_{k}$, and $A^{c}$ is the complement of A. $p_{i}$ and $p_{j}$ are the probabilities of a certain target in i and j compound’s target profiles respectively.

### Functional Enrichment Analysis and Biological Network Construction

To investigate the potential signaling pathways or biological processes regulated by GZFLC and each herb, functional enrichment analysis was carried out in Comparative Toxicogenomics database (Davis et al., 2019) to obtain the over represented Gene Ontology (GO) biological processes (BP)/KEGG signaling pathways based on the potential targets of GZFLC and each herb. The significantly enriched GO BP/KEGG signaling pathways with *p* < 0.05 after Benjamin’s correction were selected for further study. All significantly enriched GO BP/KEGG signaling pathways were categorized by means of key words mapping.

The herbs-compounds-biological functional modules-biological molecules network of GZFLC was constructed to elucidate its putative anti-primary dysmenorrhea mechanisms by considering protein–protein interactions and crosstalk among pathways. First, herbs were connected to their representative compounds. Second, the potential targets of GZFLC were mapped into protein–protein interaction network and the targets were annotated by its biological functions. Third, an edge was added between herbs and biological functional modules if potential targets of a certain herb were significantly enriched in biological functional modules related GO BP or KEGG signaling pathways (*p* < 0.05). The herbs-compounds-biological functional modules-biological molecules network was visualized using Cytoscape v3.7.2 (Shannon et al., 2003).

### Hierarchical Clustering Analysis of Anti-Primary Dysmenorrhea Drugs and Compounds in GZFLC

We hypothesized that the drugs with similar target profiles predicted by drugCIPHER-CS may have similar biological activities. The resemblance of compounds’ and drugs’ biological activities could be further defined on the basis of the similarity between concordance scores of their target profiles. Hierarchical clustering analysis of target profiles was applied to measure the biological activity resemblance of compounds and drugs. Thus, to identify anti-primary dysmenorrhea candidate compounds, hierarchical clustering analysis was conducted using R statistic software (R version 3.6.3) based on target profiles of FDA-approved anti-primary dysmenorrhea drugs and 51 compounds of GZFLC.

### COX2 Activity Assay

As GZFLC had multiple compounds and targets, we first qualitatively explored the relativity of representative GZFLC compounds and targets. And then we selected predicted compounds and a specific target for preliminary analysis of their dosages and ratios as a starting point. *In vitro* enzyme activity assays were used to analyze the relationship between predicted GZFLC compounds’ different dosages and their intervention intensity on a specific target COX2, and to investigate GZFLC’s intervention intensity on COX2 under the specific ratio of GZFLC compounds which is relatively fixed in GZFLC. The content in the GZFLC (Jiangsu Kanion Pharmaceutical Co., Ltd. Jiangsu, China, No. 170601) was weighed and extracted with dimethyl sulfoxide (DMSO, 32.308:1, W/V, mg/ml) at 37°C for 30 min. The liquid was collected by centrifugation, and the supernatant was filtered through a 0.22 μ filter. The effect of GZFLC or pure chemicals including 1,2,3,4,6-penta-O-galloyl-beta-d-glucopyranose (purity (%) ≥98%, CAS: 14,937–32–7, Pubchem CID: 65238, Nanjing SenBeiJia Biological Technology Co., Ltd.), galloylpaeoniflorin (purity (%) ≥98%, CAS: 122,965–41–7, Pubchem CID: 46882879, Nanjing SenBeiJia Biological Technology Co., Ltd.), ethyl gallate (purity (%)≥98%, CAS: 831–61–8, Pubchem CID: 13250, Nanjing SenBeiJia Biological Technology Co., Ltd.) and gallic acid (purity (%)≥98%, CAS: 149–91–7, Pubchem CID: 370, Nanjing SenBeiJia Biological Technology Co., Ltd.) on COX2 activity was measured using cyclooxygenase-2 inhibitor screening kit (Beyotime, Cat. No. #S0168). Briefly, the recombinant human COX2 assay mixture (total volume = 90μL/well) consisting of assay buffer, cofactor, COX2 enzyme, and test compound (diluted in DMSO, 5.56% DMSO finally in assay) was incubated at 25°C for 5 min. Detection of the product was performed by adding probe (5μL/well). Arachidonic acid was added and the reaction was allowed to proceed for 15 min at 37°C. The fluorescence of the resulting solution was measured using SpectraMax M2e multilabel plate reader (Molecular Devices, United Kingdom). COX2 specific inhibitor ibuprofen (98.82% purity, AbMole, Cat. No. M3359) was included as a positive control. Data was represented as $\bar{X}\pm SD$ of duplicated samples. The effect of the samples on COX2 was calculated using the following equation:

$$\text{Relative activity of COX}2\left(\%\right)=\left(\frac{RFU_{\text{sample}}-RFU_{BW}}{RFU_{A}-RFU_{BW}}\right)\times 100\%.$$

### Animals

Female ICR mice (18–22 g of weight, SPF, license number: SCXK (SU) 2012–0004) were provided by the comparative medicine center of Yangzhou University. All animal experimental procedures were performed in accordance with the Guide for the Care and Use of Laboratory Animals and were approved by the animal ethics committee of the Institutional Animal Care and Use Committee (IACUC) of Kanion Pharmaceutical Co., Ltd.

### Uterine Myometrial Cells Culture

Isolation and culture of primary myometrial cells were prepared as previously reported (Mosher et al., 2013; Sun et al., 2017). Briefly, Uterine were removed from adult female ICR mice (7–8 weeks old, purchased from Nanjing Branch of Beijing Weitong Lihua Laboratory Animal Technology Co., Ltd., license number: SCXK (SU) 2016–0003). Uterine were cut longitudinally, and endometria were scraped gently. The myometrial cells were digested in 2 ml collagenase Ⅱ (GIBCO, Cat. No. 1797319) (0.025 g L^−1^), then cells were plated onto 25 cm^2^ culture bottle (Costar, US) and were cultured in complete DMEM (GIBCO, Cat. No. 11330032) at 37°C with 5%CO_2_.

### Immunofluorescence Assay

Myometrial cells were seeded in 96 well black-walled clear-bottom plates (Costar, US) (4×10^4^/ml density), and incubated overnight at 37°C in air containing 5%CO_2_. The blank group and model group were added with 100 μL serum-free medium, and the GZFLC group was added with GZFLC (Jiangsu Kanion Pharmaceutical Co., Ltd. Jiangsu, China, No. 20160319) solution prepared with serum-free medium (25 g⋅L^−1^) for 1 h. The supernatant was discarded, 100 μL serum-free medium was added to the control group, and 100 μL oxytocin (Cayman, 11,799) (final concentration of 28 μM) was added to the model group and GZFLC group for 15 min respectively. The supernatant was discarded, fixed with paraformaldehyde (Amresco, Cat. No. J531) (0.4 g⋅L^−1^) for 15 min, washed with PBS for 5 min 3 times, and blocked with 5% goat serum (Boster, Cat. No. 10E12B09)/0.3% tritonx-100 for 1 h. Then cells were incubated with primary antibody against p-SAPK/JNK(CST), p-p44/42 MAPK(Erk1/2) (CST), p-p38(CST) (1:150, 1:150, 1:2000) at 4°C overnight. The next day, it was equilibrated to room temperature, followed by 1 h incubation with Alexa Fluor^®^ 488 Conjugate (1:1000, Invitrogen, United States) at room temperature, washed 3 times with PBS, counterstained with DRAQ5^®^ for 5 min, washed once with PBS, and imaged with a high-content instrument (Thermo, ArrayScanⅦ).

### Oxytocin-Induced Dysmenorrhea in Mice

Oxytocin-induced dysmenorrhea in mice was performed according to the method previously reported (Sun et al., 2002). Fifty female ICR mice (license number: SCXK (SU) 2012–0004) were evenly divided into five groups as follows: normal group, model group, ibuprofen group and the GZFLC groups of different doses (0.54 g/kg and 1.08 g/kg). Except for the normal group (subcutaneous injection of saline), the other groups were administrated by subcutaneous estradiol benzoate injection (0.05 ml/mouse on the first and 10th day, and 0.025 ml/mouse on the remaining days) for ten consecutive days. The model group, GZFLC groups, and Ibuprofen group were orally administrated on the fourth day with 0.5% CMC-Na of the same volume (20 ml/kg), GZFLC (0.54 g/kg and 1.08 g/kg) and ibuprofen (0.1 g/kg) for seven days (once a day) in the period, respectively. On the 10th day, oxytocin (0.2 ml/2 IU/mouse) was administered by peritoneal injection 1 h after the last administration. The number of animal writhing times was observed and recorded within 30 min after injection of oxytocin. The number of writhing inhibition rate (IR %) was calculated according to the formula (inhibition rate% = (number of writhing times in the model group-number of writhing times in the medication group)/number of writhing times in the model group) × 100%). The mice were sacrificed immediately. The uterus tissues were fixed with 10% formalin for further study.

### Pathological Examination

The uterus tissues were fixed in 10% formalin and stained with hematoxylin and eosin (H&E). Histopathological changes were observed by optical microscope (OLYMPUS DX45, Japan) including degeneration and necrosis in the endometrial epithelial cells, congestion, edema, inflammatory cell infiltration in the lamina propria, the number of glands (increased or decreased), secretion in the gland cavity, and lesions in the muscle layer and serosal layer. According to the severity of the lesion, it was recorded as one point (mild), two points (moderate), three points (severe), four points (extremely severe), no lesion was recorded as 0 point. All scores were accumulated and the average score ($\bar{X}\pm SD$) of each animal in each group was calculated.

### Statistical Analysis

The Fisher’s exact test was used to identify significantly enriched KEGG signaling pathways and GO biological processes. The immunofluorescence assay results were analyzed by SPSS 13.0 software. One-way and two-way analysis of variance (ANOVA) were used to calculate statistical levels between groups. The enzyme activity assay results were analyzed using Graphpad Prism software (Graph Pad, United States) presented as $\bar{X}\pm SD$ of duplicated samples. The animal assay results were analyzed using Graphpad Prism software (Graph Pad, United States) presented as $\bar{X}\pm SEM$ or $\bar{X}\pm SD$ of duplicated samples. *p*-value less than 0.05 was considered to be statistically significant.

## Results

### Targets Prediction and Validation of GZFLC Compounds

**FIGURE 2:**
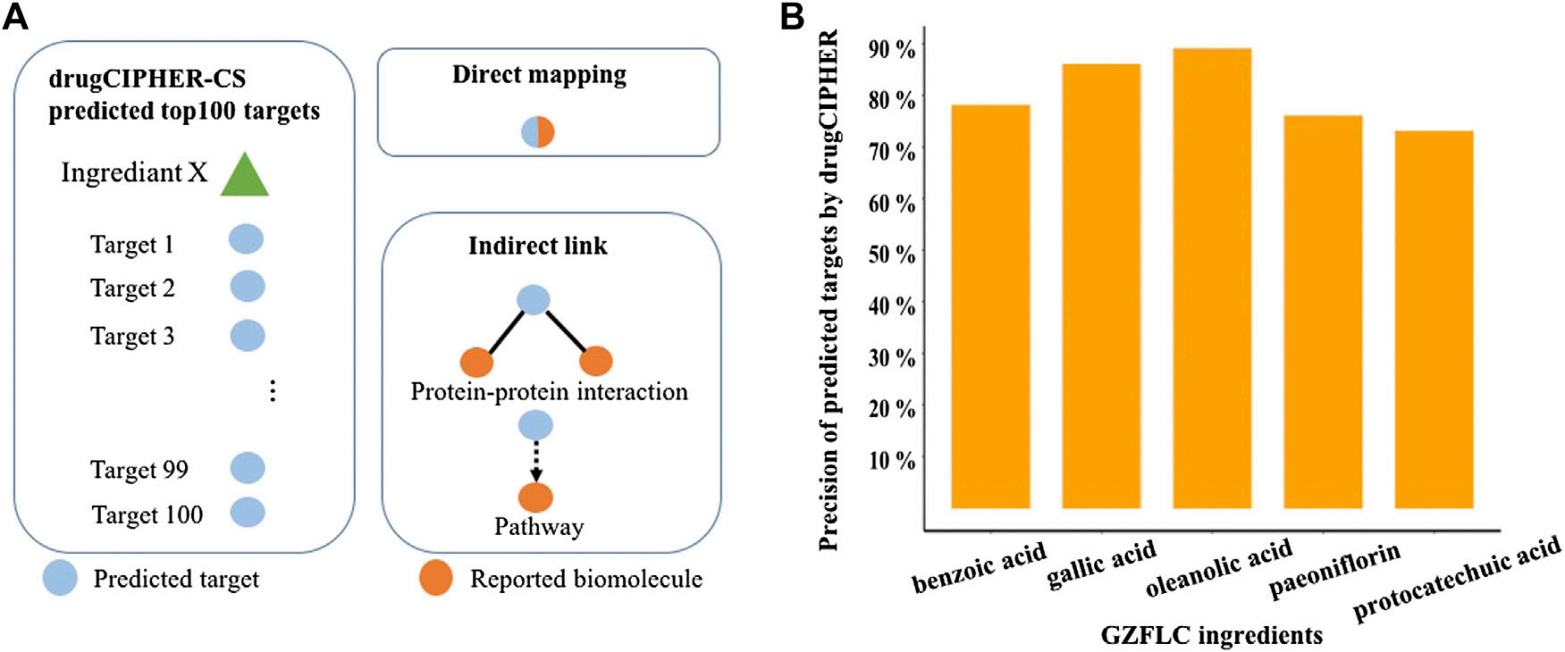
**Target prediction of GZFLC compounds and literature validation (A)** Top 100 predicted targets were considered as the target profile of each compound. Direct mapping represents that the predicted targets are the same as reported biomolecules. Indirect link represents that the predicted targets are connected to reported biomolecules through protein–protein interactions or pathways **(B)** Reliability validation of predicted targets of representative compounds in GZFLC.

We selected 51 representative compounds of GZFLC according to our previous investigations to further study. It has been known that bioactive natural compounds may have multiple targets to exert certain therapeutic effects. To reveal the pharmacological mechanisms of GZFLC for treating primary dysmenorrhea, potential targets of 51 representative compounds were predicted by our state-of-art network-based algorithm drugCIPHER-CS due to the lack of these compounds’ known target records. The top 100 predicted targets of each compound were selected as its potential targets for high precision in this algorithm, called target profiles of each compound. Moreover, the reliability of the predicted targets was verified making use of literature mining method based on text searching. The predicted targets were validated by the reported targets with literature evidence via direct mapping or indirect link as shown in Figure 2A. The precision of predicted targets was used to measure the reliability, which stands for the percentage of the predicted targets supported by literatures. Greater than 73% of the predicted targets of compounds could be supported with literature evidence in Figure 2B, while the others could be novel targets which would be investigated in the future study. These results indicated the reliability of our predicted targets of GZFLC compounds used for further investigation of pharmacological mechanisms.

### Network Target Analysis of Anti-Primary Dysmenorrhea Mechanisms of GZFLC

At first, there was a hypothesis that if a biological molecule was an important target in the pharmacological effects of GZFLC, it may rank top or appear in the target profiles of many compounds in GZFLC. Then, based on this hypothesis, we selected the most representative targets of GZFLC compared to the null model with Poisson binomial statistics as potential targets regulated by GZFLC to uncover its therapeutic mechanisms for primary dysmenorrhea. Finally, we got 240 potential targets as GZFLC network target and made functional enrichment analysis on these targets. The significantly enriched biological processes and signaling pathways were achieved as listed in Table 1. According to key words mapping, the network target of GZFLC significantly enriched in some pain, inflammation, endocrine, blood circulation and energy metabolism related biological processes and signaling pathways (*p* < 0.05). Hence, potential functional modules regulated by GZFLC on primary dysmenorrhea could be related to pain, inflammation, endocrine, blood circulation and energy metabolism.

**TABLE 1:** Several enriched biological processes and signaling pathways predicted to be regulated by GZFLC using functional enrichment analysis.

Class	Biological processes and signaling pathways	*p*-value
Pain	cAMP signaling pathway	8.80E-14
	MAPK signaling pathway	1.12E-10
	cGMP-PKG signaling pathway	5.75E-10
	HIF-1 signaling pathway	1.42E-08
	Sensory perception of pain	4.37E-05
	Muscle contraction	7.28E-05
	Regulation of neurotransmitter levels	2.74E-03
	Regulation of cytosolic calcium ion concentration	5.64E-03
Inflammation	Cytokine-mediated signaling pathway	8.65E-20
	Inflammatory response	4.43E-14
	Inflammatory mediator regulation of TRP channels	8.90E-05
	NF-kappa B signaling pathway	1.20E-03
Endocrine	Steroid hormone mediated signaling pathway	1.39E-23
	Estrogen signaling pathway	8.25E-18
	Regulation of hormone levels	1.34E-17
	Prolactin signaling pathway	1.36E-13
	Oxytocin signaling pathway	1.79E-05
	GnRH signaling pathway	1.58E-02
	Steroid hormone biosynthesis	2.21E-02
Blood circulation	Blood circulation	9.66E-18
	VEGF signaling pathway	1.63E-06
	Platelet activation	3.01E-06
	Blood coagulation	3.26E-06
	Regulation of blood pressure	1.73E-05
	Blood vessel development	4.37E-04
Energy metabolism	PPAR signaling pathway	2.01E-13
	ATP metabolic process	8.81E-09
	AMPK signaling pathway	4.53E-09

**FIGURE 3:**
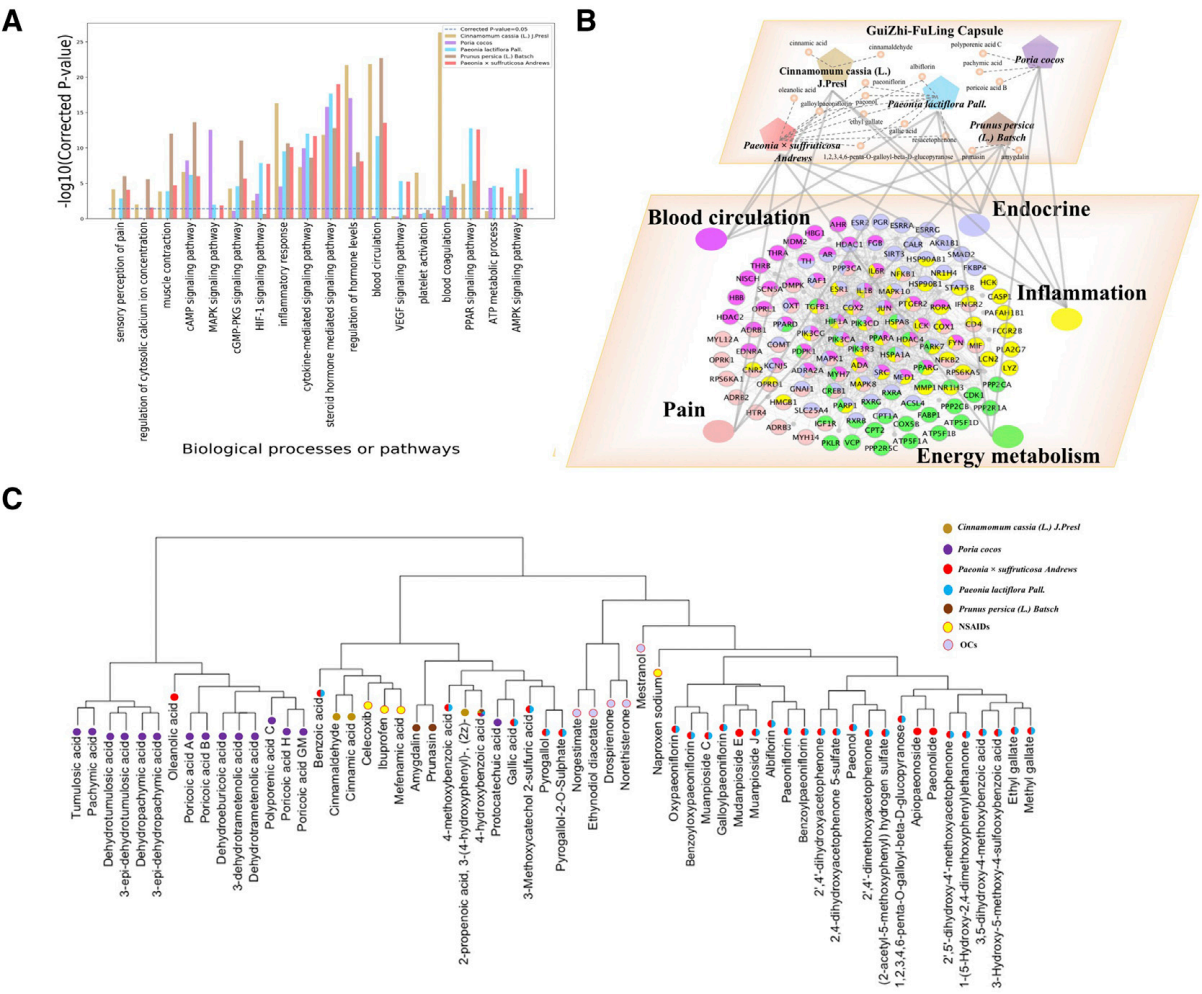
**(A)Functional enrichment analysis of each herb’s potential targets (B)Herbs-compounds-biological functional modules-biological molecules network to depict the underlying biological mechanisms of GZFLC (**Different color nodes represent different function module and its related biomolecules, namely, fuchsia nodes: Blood circulation function module and its related biomolecules; lavender nodes: Endocrine function module and its related biomolecules; yellow nodes: Inflammation function module and its related biomolecules; green nodes: Energy metabolism function module and its related biomolecules; pink nodes: Pain function module and its related biomolecules.) **(C)Hierarchical clustering tree of GZFLC compounds and current anti-primary dysmenorrhea drugs.**

We also got the potential targets of each herb in GZFLC respectively and conducted the functional enrichment analysis for these targets of each herb with the significantly enriched biological processes and signaling pathways of GZFLC as shown in Figure 3A. Those results could help us to better elucidate effects of GZFLC for treating primary dysmenorrhea. *Cinnamomum cassia (L.) J. Presl*, *Prunus persica (L.) Batsch* mainly regulated pain, inflammation, endocrine, blood circulation modules. *Paeonia lactiflora Pall.* and *Paeonia × suffruticosa Andrews* mainly regulated pain, inflammation, endocrine, blood circulation and energy metabolism modules. *Poria cocos* mainly regulated inflammation, endocrine and energy metabolism modules. To further elucidate the underlying molecular mechanisms of GZFLC, the biological molecular network targeted by GZFLC was constructed to uncover the relationships among herbs, compounds, biological functional modules and targets in Figure 3B. These results suggested that GZFLC could reduce pain and inflammation, and improve endocrine, blood circulation and energy metabolism, which was supported by evidences from the following *in vitro* and *in vivo* experiments.

Moreover, the hierarchical clustering analysis of GZFLC compounds and first-line drugs for primary dysmenorrhea was performed to further investigate the anti-primary dysmenorrhea activities of GZFLC compounds. The therapeutic effects of first-line drugs might suggest hypothetical effects for several compounds in GZFLC within the same cluster. The target profiles of GZFLC compounds and nine FDA-approved anti-primary dysmenorrhea drugs including non-steroidal anti-inflammatory drugs (NSAIDs: Ibuprofen, Naproxen sodium, Mefenamic acid and celecoxib) and oral contraceptives (OCs: Norgestimate, Ethynodiol diacetate, Drospirenone, Mestranol and Norethisterone) were predicted by drugCIPHER-CS firstly. Then, hierarchical clustering analysis was performed based on target profiles of GZFLC compounds, NSAIDs and OCs. These two kinds of first-line drugs have different mechanisms on primary dysmenorrhea. First, NSAIDs are often taken to exert analgesic effects via inhibition of cyclooxygenase (Brune and Patrignani, 2015). The compounds of GZFLC in the same cluster with the NSAIDs might be potential active compounds to exert analgesic effects. As shown in Figure 3C, the four NSAIDs were clustered into two major groups including 36 compounds of GZFLC, indicating that these compounds might have a similar mechanism of analgesic effects to relieve primary dysmenorrhea with NSAIDs. Second, the OCs were used to regulate hormone levels to suppress ovulation, thereby reducing dysmenorrhea (Proctor et al., 2001). The compounds in GZFLC in the same cluster with OCs were considered to have a similar therapeutic mechanism on primary dysmenorrhea with OCs. As shown in Figure 3C, the five oral contraceptives were clustered with 23 compounds from GZFLC, which mainly derived from *Paeonia lactiflora Pall.* and *Paeonia × suffruticosa Andrews*, suggesting that these compounds might have the bioactivity of regulating hormone levels to improve endocrine, thereby relieving primary dysmenorrhea.

### The Underlying Mechanisms of GZFLC on Primary Dysmenorrhea

**FIGURE 4:**
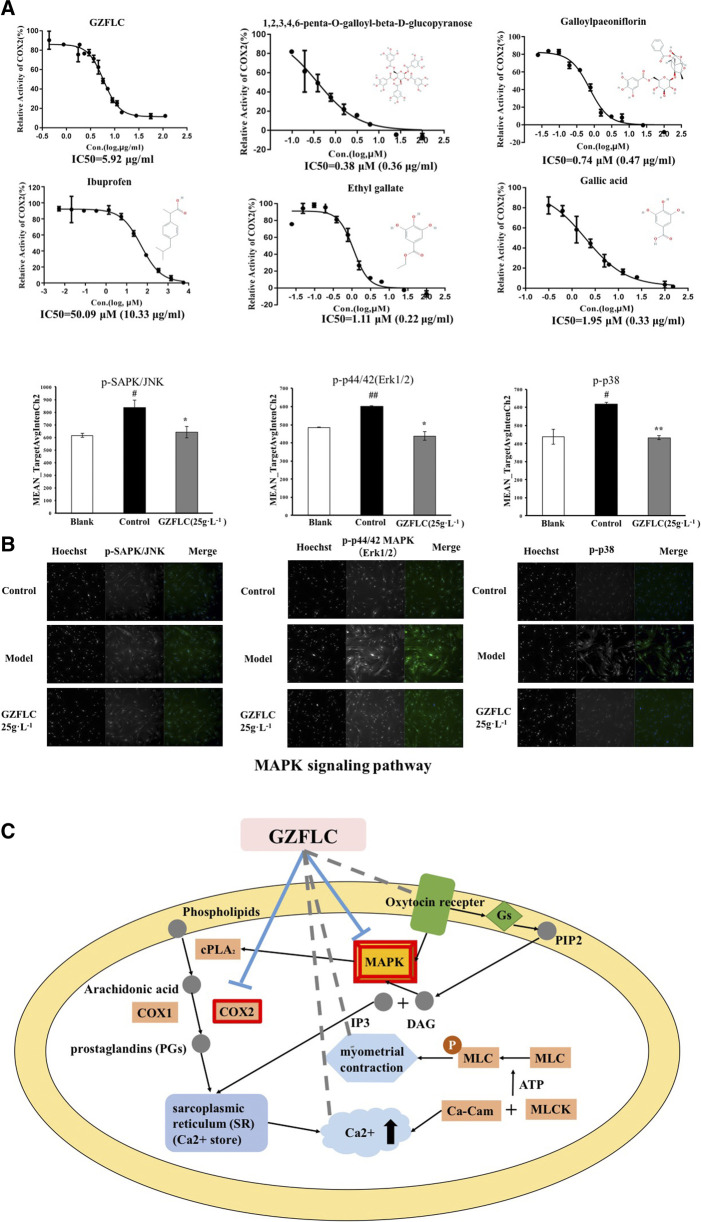
**The potential molecular mechanisms of analgesia effects of GZFLC on primary dysmenorrhea (A)** GZFLC and four of its compounds were verified to inhibit COX2 *in vitro*, ibuprofen was selected as a positive control **(B)** GZFLC was verified to downregulate predicted MAPK signaling pathway in uterine smooth muscle cells. *p < 0.05, **p < 0.01, GZFLC group compared with Model group. #*p* < 0.05, ##*p* < 0.01, Model group compared with blank group. Data was represented as $\bar{X}\pm SD$ (n = 3) **(C)**The regulatory mechanisms of analgesia effects induced by a group of compounds from GZFLC (red rectangle represents that GZFLC-targeted pathways and biological molecules are from network target analysis and have been verified by bioassays. Gray dotted line represents that GZFLC could regulate biological molecules or processes from network target analysis which were verified in literatures.)

Recent studies have demonstrated that hyper-secretion of prostaglandins and an increased uterine contractility are two causes of pain associated with dysmenorrhea (Bernardi et al., 2017). NSAIDs exert analgesia effects to effectively alleviate primary dysmenorrhea through decreasing prostaglandin levels via inhibition of cyclooxygenase-mediated production (Marjoribanks et al., 2010). COX2 is an important cyclooxygenase that catalyzes arachidonic acid conversion to prostaglandins, which is a major target of NSAIDs. Meanwhile, COX2 was predicted as an important biomolecule regulated by GZFLC from our network target analysis. We therefore examined whether GZFLC and its potential bioactive compounds above could affect COX2 activity. We found that GZFLC and four predicted main compounds, including 1,2,3,4,6-penta-O-galloyl-beta-d-glucopyranose, galloylpaeoniflorin, ethyl gallate and gallic acid, could inhibit the activity of COX2 *in vitro*. As shown in Figure 4A, GZFLC inhibited the activity of COX2 *in vitro* with an IC50 value of 5.92 μg/ml 1,2,3,4,6-penta-O-galloyl-beta-d-glucopyranose, galloylpaeoniflorin, ethyl gallate and gallic acid inhibited the activity of COX2 *in vitro* with IC50 values of 0.38 μM (0.36 μg/ml), 0.74 μM (0.47 μg/ml), 1.11 μM (0.22 μg/ml) and 1.95 μM (0.33 μg/ml) respectively. Ibuprofen was selected as the positive control in our study and the results showed that ibuprofen potently inhibited the activity of COX2 with an IC50 value of 50.09 μM (10.33 μg/ml).

As a result, the network target of GZFLC was significantly enriched in MAPK signaling pathway. MAPKs could increase cPLA2 activity and hence result in prostaglandin production and myometrial contraction (Lin et al., 1993). Therefore, we used the immunofluorescence assay to determine whether GZFLC could affect MAPK signaling pathway to reduce myometrial contraction. We found that uterine smooth muscle cells in oxytocin-induced dysmenorrhea murine model displayed the increase of p-SAPK/JNK, p-p44/42 MAPK (Erk1/2) and p-p38 protein levels, and this effect was attenuated in cells pre-treated with GZFLC (25 g⋅L^−1^) as shown in Figure 4B, which suggested that GZFLC could downregulate the MAPK signaling pathway.

Integrating network target analysis and *in vitro* experiments, as shown in Figure 4C, GZFLC could inhibit the activity of COX2 and downregulate MAPK signaling pathway to reduce the production of PGs and myometrial contraction to exert analgesia effects for treating primary dysmenorrhea. In addition, GZFLC could regulate cytosolic calcium ion concentration, intervene oxytocin receptor (OTR) to reduce myometiral contraction and decrease inflammation, which was validated in literatures (Sun et al., 2016a; Zhong et al., 2016b) and subsequent *in vivo* experiments.

Primary dysmenorrhea is also related to imbalances in women’s endocrine system during the menstrual cycle (Britannica, The Editors of Encyclopedia, 2016). And a color Doppler study (Altunyurt et al., 2005) has found that there was increased impedance to blood flow within the uterus of patients with primary dysmenorrhea on the first day of the menstrual cycle, suggesting the disturbance of blood circulation in primary dysmenorrhea patients. Meanwhile, a previous study (Xiong et al., 2019) has found that there were disorders of energy metabolism in primary dysmenorrhea rats. Thus, Blood circulation, endocrine and energy metabolism may be three important functional modules to be regulated to gain sustained beneficial effects. According to the network target analysis, GZFLC was predicted to regulate blood circulation, endocrine and energy metabolism-related biological processes and signaling pathways, which may contribute to the sustained beneficial effects of GZFLC on primary dysmenorrhea. The effect of GZFLC on blood circulation, endocrine and energy metabolism have been verified in literatures (Su et al., 2015; Sun et al., 2016a; Xiong et al., 2019).

### Effects of GZFLC on Primary Dysmenorrhea in Oxytocin-Induced Murine Model

**FIGURE 5:**
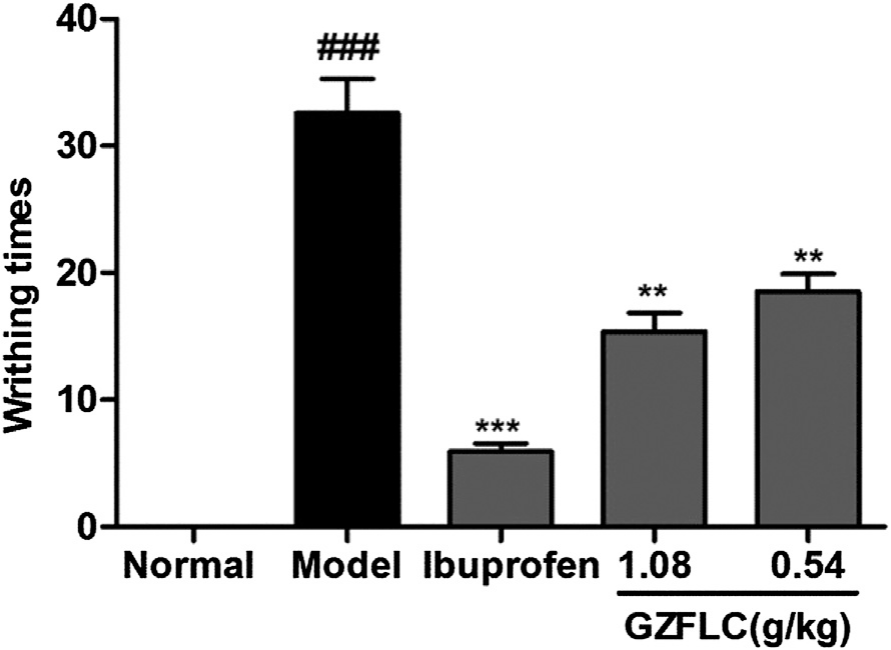
**Effects of GZFLC on writhing response in oxytocin-induced dysmenorrhea murine model.** ICR mice were divided into five groups as follows: normal group, model group (0.5% CMC-Na, 20 ml/kg), ibuprofen (0.1 g/kg) and the GZFLC groups of different doses (0.54 g/kg and 1.08 g/kg). The number of animal writhing times was observed and recorded within 30 min after injection of oxytocin. **p < 0.01, ***p < 0.001, GZFLC group compared with Model group. ###*p* < 0.001, Model group compared with normal group. Data was represented as $\bar{X}\pm SEM$ (n = 10).

**FIGURE 6:**
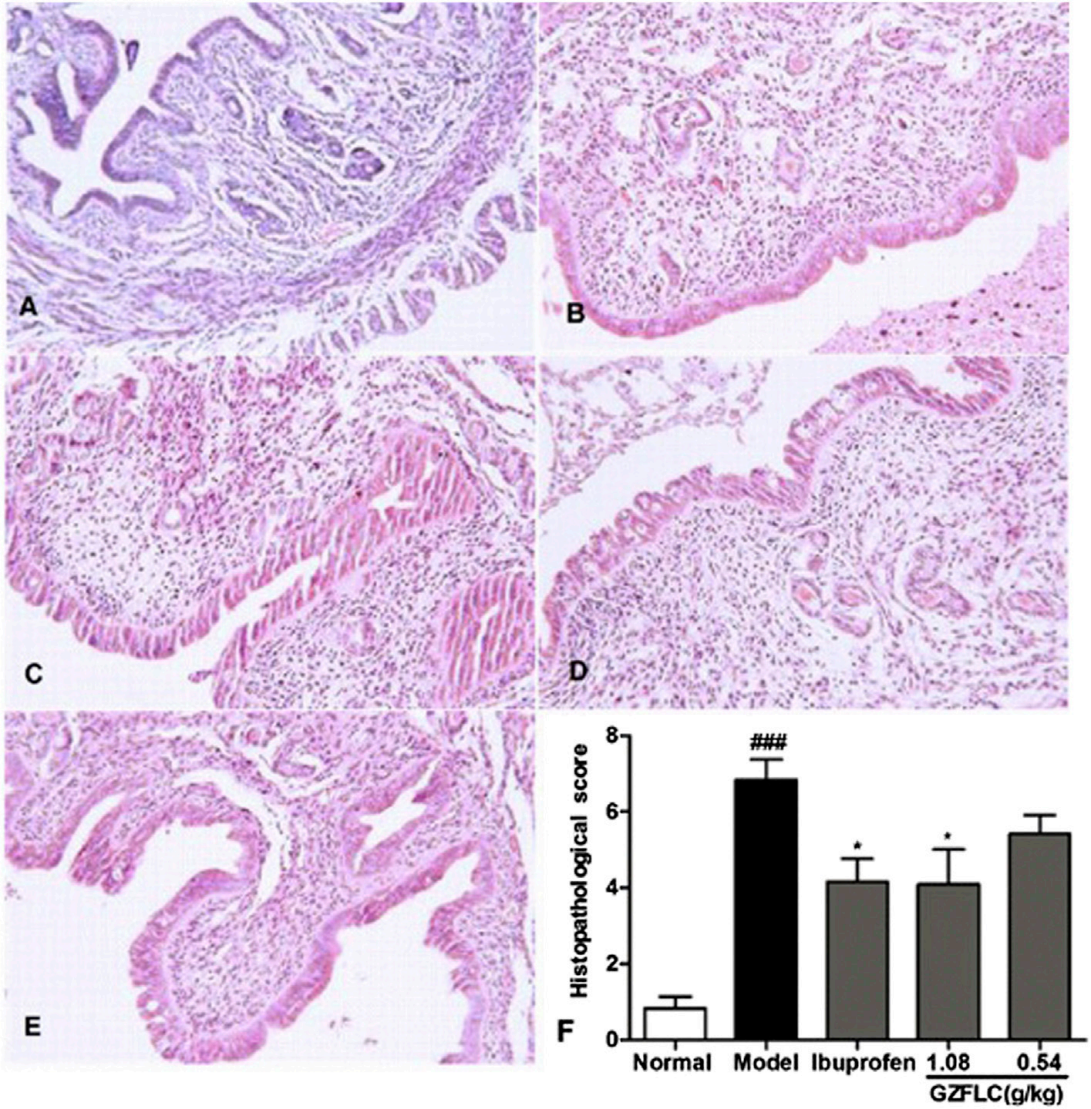
**(A-E) Photomicrography of the uterine tissues with H&E staining in normal group, model group, ibuprofen (0.1 g/kg), GZFLC groups (1.08 g/kg and 0.54 g/kg) respectively (F) Effects of GZFLC on uterine histopathology in oxytocin-induced dysmenorrhea murine model.** *p < 0.05, GZFLC group compared with Model group. ###*p* < 0.001, Model group compared with normal group. Data was represented as $\bar{X}\pm SD$ (n = 6).

We have investigated the effect of GZFLC on the writhing response in oxytocin-induced dysmenorrhea murine model to study its analgesic activity. Analgesic activity was determined by observed decreases of writhing times in oxytocin-induced murine model, which was a method for quantitative evaluation of pain. As shown in Figure 5, GZFLC significantly inhibited the oxytocin-induced writhing response. The inhibition percentages of writhing times were 43.3% and 52.8% after oral administration of GZFLC (0.54 g/kg and 1.08 g/kg) respectively while 85.5% after oral administration of ibuprofen. The numbers of writhing times at GZFLC groups (0.54 g/kg and 1.08 g/kg) and ibuprofen group (0.1 g/kg) were 18.5 ± 4.5, 15.4 ± 4.6 and 5.9 ± 2.1 respectively, significantly lower than that of the model group (32.6 ± 8.5). Each herbal medicine in GZFLC was of the same amount and their dosages and ratios were the same as the traditional dosages and ratios in Guizhi Fuling Wan (Committee 2015). Next, we have investigated effect of GZFLC on uterine histopathology in oxytocin-induced dysmenorrhea murine model. As shown in Figures 6A,B, the histopathological results showed that compared with the normal group, the model group mainly showed degeneration and necrosis of endometrial epithelial cells, lamina propria edema, a small amount of inflammatory cell infiltration, reduced number of glands in the lamina propria, secretions in the gland cavity, and the disordered arrangement of smooth muscle cells in the muscle layer. There was a significant difference between the model group and the normal group, indicating that the oxytocin-induced dysmenorrhea murine model was successfully constructed. Meanwhile, ibuprofen and GZFLC had the effect on reducing the degree of uterine lesions as shown in Figures 6C–E. Moreover, there was a significant difference in 1.08 g/kg of GZFLC group compared with the model group (*p* < 0.05) as shown in Figure 6F.

## Discussion and Conclusion

Guizhi-Fuling capsule, derived from a classic herbal formula, has been used for the therapy of primary dysmenorrhea for a long time in China. The clinical data has shown the analgesic and sustained beneficial effects of GZFLC on menstruating women with primary dysmenorrhea. However, the pharmacological mechanisms of GZFLC on primary dysmenorrhea are less characterized from a holistic perspective. In this study, we analyzed underlying pharmacological mechanisms of GZFLC in terms of two major clinical effects of GZFLC on primary dysmenorrhea. Generally, the network target analysis of GZFLC indicated that GZFLC may exert analgesic and sustained beneficial effects on primary dysmenorrhea through these five biological functional modules including pain, inflammation, endocrine, blood circulation, and energy metabolism.

GZFLC exerted therapeutic effects on basis of its compounds and their intervention on targets. For analgesic effect, GZFLC and four predicted compounds, including 1,2,3,4,6-penta-O-galloyl-beta-d-glucopyranose, galloylpaeoniflorin, ethyl gallate and gallic acid, were verified to inhibit COX2 activity *in vitro* through COX2 activity assays, which gave hints that 1,2,3,4,6-penta-O-galloyl-beta-d-glucopyranose, galloylpaeoniflorin, ethyl gallate and gallic acid could be used as quality control markers of GZFLC in the treatment of primary dysmenorrhea. In the meanwhile, COX2 and its production PGF2α could be used as optimizing objective and quantitative surrogate outcomes of GZFLC for primary dysmenorrhea. In addition, GZFLC was verified to downregulate MAPK signaling pathway in uterine smooth muscle cells to reduce uterine smooth muscle contraction via an immunofluorescence assay.

From perspectives of TCM, GZFLC is mainly used to treat blood stasis syndromes, and a meta-analysis of randomized clinical trials for GZFLC in the treatment of primary dysmenorrhea (Xing et al., 2019) suggested that GZFLC may be more effective on primary dysmenorrhea in a subgroup of patients with cold blood stasis syndrome, which is one of the common cold syndromes. According to our previous research (Ma et al., 2010), cold syndromes related genes acted as a pivotal part in energy metabolism, which were tightly connected with the genes of neurotransmitters, hormones and cytokines in the neuro-endocrine-immune interaction network. Interestingly, the network target analysis has found that GZFLC could reduce pain and inflammation, improve endocrine, blood circulation and energy metabolism, which were consistent with the cold syndrome related biological modules. Hence, our study about the pharmacological mechanisms of GZFLC on primary dysmenorrhea could give a potential explanation to the underlying biological basis of the subgroup results in clinical trials.

Oxytocin-induced murine model is a commonly used animal model for primary dysmenorrhea. Our study has investigated the effect of GZFLC on the writhing response, uterine histopathology in oxytocin-induced dysmenorrhea murine model. The results showed that GZFLC could significantly inhibit the writhing response in oxytocin-induced dysmenorrhea murine model. Our network target analysis results about mechanisms of anti-primary dysmenorrhea of GZFLC were partially validated by *in vivo* experiments. The pathological assay showed that high dose of GZFLC had a significant effect on reducing the degree of uterine lesions, indicating that GZFLC could decrease inflammation. These results gave support to efficacy of GZFLC on primary dysmenorrhea. Moreover, The experiment in the early study showed GZFLC markedly reduced the expression of COX2 in uterus tissue of the experimental murine models induced by oxytocin (Sun et al., 2016a). Meanwhile, the previous study showed GZFLC suppressed P42/44 MAPK phosphorylation level in the uterus tissue in PGF2α-induced model animals (Sun et al., 2016b).

In total, GZFLC could treat primary dysmenorrhea through “multi-compound, multi-target, multi-pathway” mode, which distinguished it from two kinds of first-line drugs with few targets to selectively act on. Network target derived from the multi-target nature of TCM could be preferable to analyze and reflect complicated interactions between biological molecules of the human body and chemical compounds in TCM formulae from the perspective of network (Li and Zhang 2013). There are also some limitations in this study. Firstly, some compounds in GZFLC have been ignored which may lead to bias in our study. However, we have selected important phytochemistry and absorbed compounds of GZFLC as representatives. Secondly, the exploration about relationship between the predicted compounds, targets and functional interpretation and dosages and ratios of GZFLC compounds was limited. The analysis about dosages and ratios of compounds in TCM formulae is of great significance, which has addressed lots of researchers’ attention. There also have been several research methods developed for this issue (Zhou and Su 2019), and it is urgent to develop more appropriate methods in network pharmacology to investigate this issue. We would explore this issue in more depth in the further study. Briefly, we would first clarify the dosages and ratios of GZFLC’s representative compounds using component analysis technology such as liquid chromatography-mass spectrometry (LC-MS), then we would predict those compounds’ intervention intensity on targets and integrate their comprehensive effects on interacting targets of different biological functions within the primary dysmenorrhea related biological molecular network based on quantitative network pharmacology analysis in silico.

In summary, this work adopted combination of computational and experimental methods to reveal the underlying pharmacological mechanisms of GZFLC on primary dysmenorrhea. In the network target analysis, GZFLC may act on five functional modules of pain, inflammation, endocrine, blood circulation and energy metabolism. Next, we found that GZFLC significantly inhibited the writhing response and reduced the degree of uterine lesions in oxytocin-induced murine dysmenorrhea model. Furthermore, GZFLC may partially alleviate primary dysmenorrhea by inhibiting COX2 and downregulating MAPK signaling pathway *in vitro*. Thus, GZFLC presented pain relief and sustained benefits for primary dysmenorrhea. The results of this study showed that integrating TCM Network pharmacology and several different experimental methods could effectively elucidate underlying biological molecular mechanisms of GZFLC and identify part of the bioactive compounds. This study could provide a scientific approach for deciphering the pharmacological mechanisms of TCM herbal formulae and developing potential optimizing objective and quantitative surrogate outcomes of GZFLC for primary dysmenorrhea through network pharmacology.

## Data Availability

The raw data supporting the conclusions of this article will be made available by the authors, without undue reservation.
