# Supplementary figures and images for: Deciphering the Pharmacological Mechanisms of Guizhi-Fuling Capsule on Primary Dysmenorrhea Through Network Pharmacology
Source: Front Pharmacol. 2021 Mar 3;12:613104. doi: 10.3389/fphar.2021.613104 (PMC7966503; doi:10.3389/fphar.2021.613104)

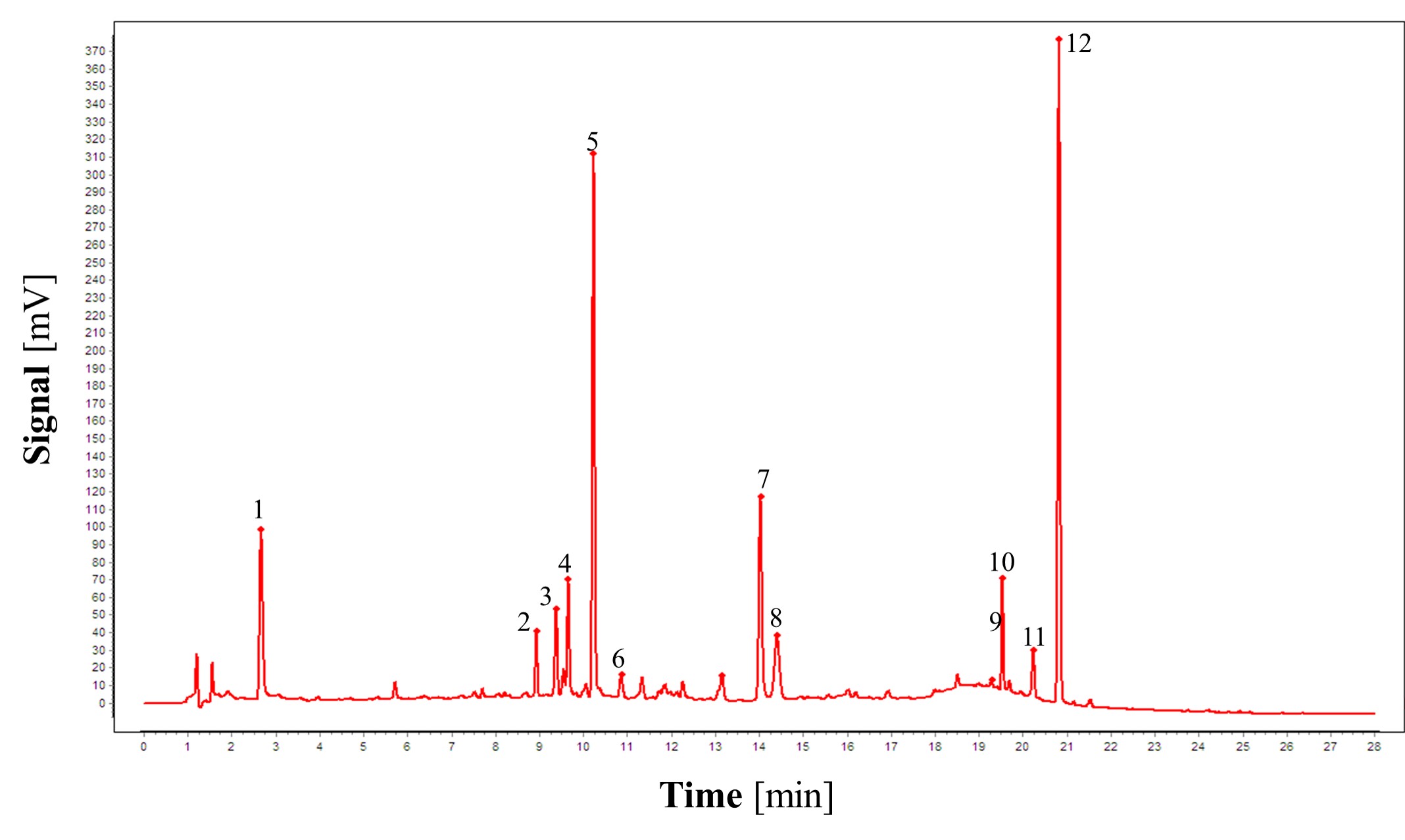

Supplement: Supplementary file 1 [file image1.jpeg]
